# Experiences of Newborn Fathers in Supporting Breastfeeding Based on the Social–Ecological System: A Qualitative Study

**DOI:** 10.1155/jonm/5341647

**Published:** 2026-06-28

**Authors:** Siya Lin, Yacui Huang, Shunmei Chen, Minyan Wu, Haiai Long, Dan Tian

**Affiliations:** ^1^ Neonatal Intensive Care Unit, Zhongshan Hospital, Xiamen University, Xiamen, China, xmu.edu.cn; ^2^ Obstetrics and Gynecology Medical Center, Zhujiang Hospital, Southern Medical University, Guangzhou, China, fimmu.com

**Keywords:** breastfeeding, experience, father support, newborns, nursing care, qualitative study, social–ecological system

## Abstract

**Background:**

Despite the irreplaceable benefits of breastfeeding, China’s exclusive breastfeeding rate among infants under 6 months remains far below the WHO target of 50%. Fathers play a critical role in breastfeeding outcomes, yet research exploring their experiences and challenges remains limited.

**Aim:**

To explore the experience of newborn fathers in supporting breastfeeding and provide a reference for developing an effective breastfeeding support system.

**Methods:**

Based on the social–ecological system, this descriptive qualitative study was conducted from October to December 2024. Newborn fathers from the obstetrics department of a tertiary general hospital in Guangzhou were purposively sampled (*N = *13) for semistructured interviews. Data were organized and analyzed using directed content analysis.

**Results:**

A total of three themes and seven subthemes were extracted, including microsystem (newborn fathers’ recognition and breastfeeding support but insufficient self‐efficacy), mesosystem (the need for optimization of family support systems), and macrosystem (the need for improvement in sociocultural support systems).

**Conclusion:**

Newborn fathers recognize and support breastfeeding, but their low self‐efficacy results in inadequate supportive behaviors. Multiple factors, such as individual knowledge and attitudes, family and workplace support, sociocultural influences, and public service systems, interact and influence each other.

**Implications for Nursing Management:**

Effective strategies to narrow fathers’ attitude–behavior gap in breastfeeding include practical skill training, continuous daily support, family communication enhancement, workplace optimization, sociocultural guidance, and improved public services. Strengthening research and advocacy on paternal roles can further reduce fathers’ burden and increase their participation, thereby promoting breastfeeding practices.

## 1. Background

Breastfeeding is universally recognized as a cornerstone of infant health and development, offering irreplaceable benefits such as enhanced immunity, optimal nutrition, and strengthened maternal–infant bonding, and even has a small positive effect on IQ in later childhood [1]. Despite decades of global advocacy, exclusive breastfeeding rates remain suboptimal in many regions. In China, only 29.2% of the infants under six months are exclusively breastfed [2], falling significantly short of both the WHO’s 50% target and the national objective outlined in China’s National Programme of Action for Child Development 2021–2030 [3, 4]. This discrepancy highlights systemic challenges in translating policy into practice, especially sociocultural, familial, and institutional barriers. While maternal factors, such as lactation difficulties, postpartum recovery, and workplace constraints, are well documented, the role of fathers in breastfeeding support remains underexplored, despite their critical influence on maternal decision‐making and infant care [5]. China’s Breastfeeding Promotion Action Plan (2021–2025) calls for enhanced family and societal support to improve breastfeeding rates [6]. However, implementation efforts remain predominantly maternal centric.

Paternal influence on breastfeeding practices is well established. Sherriff et al. [7] conceptualized paternal support as encompassing five dimensions: knowledge, positive attitude, involvement in the decision‐making, practical support, and emotional support. Empirical evidence corroborates this framework. For instance, a national survey by the China Development Research Foundation [2] revealed that infants with supportive fathers exhibit significantly higher exclusive breastfeeding rates than those without paternal engagement. A multicenter cross‐sectional study in Southeast China indicates that high‐level father support for breastfeeding self‐efficacy effectively increased the exclusive breastfeeding rate at 6 weeks postpartum [8]. Similarly, Koksal et al.’s [9] systematic review demonstrated that paternal involvement enhances breastfeeding outcomes and maternal self‐efficacy. Nevertheless, Bogale et al. [10] highlight persistent gaps: fathers’ participation in feeding children aged 6–24 months remains low globally, underscoring the urgent need for targeted health education.

However, while this quantitative evidence robustly establishes the correlation between paternal support and positive outcomes [8, 11], it provides limited insight into the subjective, lived experiences that underlie these supportive behaviors. In the Chinese context, it remains largely unexplored how fathers perceive their role, what emotional and practical challenges they face, and how they interpret and interact with family, work, and cultural influences. This methodological gap necessitates qualitative inquiry to uncover the subjective dimensions of paternal engagement.

In recent years, qualitative inquiry has begun to delve into fathers’ experiences of supporting breastfeeding within specific contexts. For example, employing a phenomenological approach, the study by Li et al. [12] explored the lived experiences of fathers of preterm infants in supporting breastfeeding and identified multiple challenges encountered in the process. However, this research focuses predominantly on high‐risk, medically dominated extreme situations. For example, newborns who need to be admitted to the neonatal intensive care unit for various reasons, such as premature birth or congenital malformations. In contrast, the “normative postpartum context”—characterized by rooming‐in and a family‐centered early adaptation phase, which encompasses the vast majority of families with healthy, term newborns—has not been accorded commensurate in‐depth and systematic scrutiny. It remains substantially underexplored how fathers perceive, experience, and enact their supportive roles in this setting, including the challenges they face, the resources they mobilize, and their interactive dynamics within social–ecological systems (SESs).

The SES offers a robust analytical framework for such exploration. It describes how environmental, societal, institutional, interpersonal, and individual factors shape the actions and health behaviors of individuals [13]. This theory posits that dynamic interactions across three interconnected systems shape human development: (1) microsystem: individual‐level factors (biological, psychological, and social); (2) mesosystem: intermediate social networks (family, workplace, and peers); and (3) macrosystem: societal structures (cultural norms, institutions, and policies) [13]. In China, this theory has demonstrated adaptability across diverse health contexts, including chronic disease management and mental health interventions [14–17]. Given that paternal breastfeeding support is influenced by factors ranging from personal confidence to workplace policies and cultural norms, the SES framework provides a structured lens to examine these multilevel interactions systematically. For example, workplace flexibility (macrosystem) affects a father’s time availability at home (mesosystem), which in turn shapes his hands‐on experience and confidence in assisting breastfeeding (microsystem). This dynamic interaction moves beyond listing barriers and facilitators, showing how interventions at one level can cascade across others and offering a comprehensive theoretical basis for multilevel support strategies.

Therefore, this study employs a qualitative approach guided by SES to examine newborn fathers’ systematically lived experiences in breastfeeding support. By elucidating the interplay of personal, familial, and societal forces, we aim to formulate targeted interventions that empower fathers as active collaborators in breastfeeding practices, ultimately advancing national efforts to achieve sustainable improvements in breastfeeding rates.

## 2. Methods

### 2.1. Design

We adopted a descriptive, qualitative study using a semistructured interview method.

### 2.2. Sample

The participants were recruited using a purposeful sampling strategy with maximum variation. Variation was sought across fathers’ age, occupation, and parenting experience to capture a wide spectrum of perspectives. Spouses of postpartum women admitted to the obstetrics department of a tertiary hospital in Guangzhou, China, were recruited as participants between October and December 2024. Inclusion criteria comprised (1) newborn fathers aged 18–60 years and (2) absence of cognitive or communicative impairments. Exclusion criteria were (1) maternal contraindications to breastfeeding (e.g., inverted nipples and hypoplastic breasts); (2) mother–infant separation (e.g., neonatal intensive care unit admission); and (3) newborns with congenital feeding barriers (e.g., cleft lip) or sucking dysfunction. These groups were excluded because our study aimed to explore paternal experiences within the context of routine, direct breastfeeding at home. The aforementioned conditions introduce specialized medical care, feeding methods (e.g., tube feeding), and support dynamics that differ fundamentally from the typical postpartum breastfeeding journey, thus constituting a distinct phenomenon outside the scope of this inquiry. Recruitment continued until data saturation was achieved.

### 2.3. Data Collection

Guided by the research objectives, a systematic literature review was conducted to inform the development of the interview protocol. Following iterative discussions within the research team, a preliminary interview guide was formulated. To enhance content validity and participant comprehension, two newborn fathers participated in pilot interviews, with their feedback informing subsequent revisions to the final interview protocol. The semistructured guide encompassed eight core domains: (1) Could you tell me about how you are feeding your baby at the moment? (2) What are your personal thoughts on breastfeeding? What do you see as its main benefits or challenges, from your perspective? (3) Please talk about your understanding of breastfeeding knowledge. What kinds of things do you feel you know about, or wish you knew more about? (4) How did you help your partner to breastfeed? Could you describe the situation and what you did? (5) In your view, why is it important, or not, for a father to learn about breastfeeding? (6) Looking ahead, what do you think might be the biggest hurdles to keeping breastfeeding going for 6 months? (7) From what you have experienced, what are the most helpful ways a father can support a breastfeeding mother?

Data were collected through one‐on‐one semistructured interviews conducted face‐to‐face with the participants. The researchers scheduled the interview time with the participants ahead of time and explained the study’s purpose and process to them before the interview. They also informed the participants about the confidentiality principles and obtained informed consent. Fathers were interviewed during the routine postpartum follow‐up visit at 42 days after delivery in an independent and quiet demonstration room/multifunction room, with a “Do Not Disturb” sign hung on the door during the interview.

During the interview, the researcher followed the interview guide, using open‐ended questions and probing techniques (e.g., “Could you give me a specific example of that?” or “How did you feel when that happened?”) to encourage participants to elaborate on their personal experiences and feelings. They also observed and recorded the participants’ expressions, actions, and psychological states, striving to ensure that the data fully, accurately, and authentically reflected the participants’ viewpoints. The interviews lasted for 20–30 min, and with the participants’ consent, the entire process was recorded, and field notes were taken.

### 2.4. Data Analysis

Data collection and analysis proceeded concurrently until thematic saturation was reached, which was determined when no new relevant themes emerged from three consecutive interviews. Within 24 h after the interview, two researchers independently transcribed the recorded content and verified any ambiguous parts with the interviewees to produce the final transcripts. Using the SES as a framework, this study employed directed content analysis, which allows for systematic identification of themes directly from interview data while using the SES framework as an initial coding structure, thereby balancing theoretical grounding with openness to emergent findings. Two researchers independently analyzed the data using NVivo 12.0 software. The specific steps included (1) defining the unit of analysis by dividing sentences reflecting the influencing factors of fathers’ support for breastfeeding into the smallest units, forming the analytical units; (2) reading the original data in depth and repeatedly understanding the content; (3) establishing a classification outline based on the SES Theory Model, categorizing the analytical units into three broad categories: micro, meso, and macro; (4) conducting content coding and classification using an open coding method, annotating important ideas and concepts in the data, and grouping similar codes to form themes and subthemes; and (5) interpreting and analyzing the results, correlating the data with the findings, and extracting corresponding examples for elaboration.

To ensure the accuracy and credibility of the data analysis results, two researchers trained in qualitative research independently conducted data extraction and analysis. In case of any disagreement on theme extraction during the analysis process, the research team would hold collective discussions and confirm the coding content with the research subjects, ultimately arriving at a reasonable interpretation and summary.

### 2.5. Ethical Considerations

This study adhered to the Declaration of Helsinki. Ethical approval was obtained from the Institutional Review Board (no. 2022‐KY‐234‐01), and written informed consent was secured from all participants.

## 3. Results

Based on the SES, the experience of newborn fathers supporting breastfeeding can be summarized into three themes and seven subthemes.

### 3.1. Demographic Characteristics of Participants

A total of 13 participants were enrolled, with demographic characteristics summarized in Table 1

**TABLE 1:** Demographic characteristics of participants (*N* = 13).

Participant no.	Age (years)	Education	Ethnicity	Residence	Occupation	Attended training (yes/no)	Parenting experience (yes/no)
F1	25	Associate Degree	Han	Urban	Clerk	No	No
F2	35	Master’s Degree	Han	Urban	Clerk	No	Yes
F3	38	Master’s Degree	Ethnic Minority	Urban	Clerk	No	Yes
F4	34	Doctoral Degree	Han	Urban	Physician	No	No
F5	28	Bachelor’s Degree	Han	Rural	Designer	No	No
F6	42	Bachelor’s Degree	Han	Urban	Healthcare Professional	No	Yes
F7	33	Bachelor’s Degree	Han	Urban	Engineer	No	No
F8	31	Bachelor’s Degree	Han	Urban	Engineer	No	No
F9	32	Bachelor’s Degree	Han	Urban	Finance Professional	No	Yes
F10	36	Bachelor’s Degree	Han	Urban	Clerk	Yes	No
F11	36	Bachelor’s Degree	Han	Urban	Engineer	No	No
F12	40	Master’s Degree	Han	Urban	Clerk	Yes	No
F13	37	Associate Degree	Han	Urban	Customer Service	No	Yes

### 3.2. Micro‐System‐Newborn Fathers Recognize and Support Breastfeeding but Exhibit Low Self‐Efficacy

Newborn Fathers Recognize and Support Breastfeeding but Exhibit Low Self‐Efficacy.

#### 3.2.1. Recognition and Support for Breastfeeding

Interviewed fathers universally acknowledged that breastfeeding enhances infant immunity, promotes intestinal development, facilitates maternal physical recovery, and reduces health risks such as breast‐related complications. Many emphasized breastfeeding as the most natural and contamination‐free feeding method, which not only fulfills infants’ nutritional requirements but also strengthens mother–infant emotional bonds through physical closeness. Additionally, breastfeeding was recognized as more economically advantageous than formula, effectively alleviating family financial burdens while reducing potential health risks from food additives due to its natural composition and safety. Fathers expressed strong personal endorsement of breastfeeding. Their statements reflect not only awareness but also a positive view of breastfeeding as a preferred infant feeding method.

F1: When the infant cried persistently and refused to latch, I didn’t know how to reposition the baby. I ended up stepping back and letting my wife handle it.

F2: Breast milk is good for the baby’s immune system. It’s easy for them to digest and helps the mother recover after birth. It can also help prevent issues like blocked ducts and reduce the risk of breast cancer.

F6: (The breastfeeding process) allows for close contact between the child and mother, which helps strengthen their bond.

F12: Breast milk is a natural food that reduces expenses, saving costs and alleviating financial burdens.

F7: Unlike formula, breast milk contains no additives. It probably has antibodies and other good things that are beneficial for newborns.

#### 3.2.2. Encountered Challenges and Insufficient Confidence in Supporting Breastfeeding

Fathers reported challenges and low breastfeeding confidence, mainly shown as (1) lack of feeding knowledge, (2) inadequate practical experience, and (3) concerns about infant nutrition. Most described significant challenges in the breastfeeding process and perceived exclusive breastfeeding as difficult to sustain. Furthermore, fathers unanimously acknowledged the need to learn supportive strategies to effectively assist mothers, particularly through positional adjustments and resolving infant latching difficulties. These reported difficulties consistently undermined their confidence, leading to a state of low self‐efficacy characterized by hesitation and a perceived inability to provide effective support. This diminished confidence often led to hesitancy in assisting with latching or positioning, and in some cases, withdrawal from the breastfeeding process altogether. Consequently, despite positive attitudes, fathers’ practical engagement remained limited, illustrating a critical attitude–behavior gap influenced by perceived incompetence.

F12: Breastfeeding is genuinely challenging for new parents. It might look simple, but it’s hard in practice—whether it’s about knowing what to do or other aspects, it’s quite complicated. It’s good for the baby, but sticking with it is a challenge.

F7: It (breastfeeding) really isn’t easy. The process is hard for us, especially because there are so many little details to pay attention to.

F10: I’ve read up on some things and know about positions, but without actually doing it, I still get nervous. I’m afraid I might drop the baby, yet I’m also scared to hold too tight.

F8: Understanding breastfeeding knowledge is essential. After assisting for a whole day yesterday, I realized just how much you need to know.

### 3.3. Mesosystem: Suboptimal Family Support System

#### 3.3.1. Maternal Constraints in Lactation and Temporal Resources

The paternal respondents consistently identified two primary maternal constraints: hypogalactia (insufficient milk production) and limited temporal/energetic capacity for breastfeeding. While demonstrating strong ideological support for breastfeeding, pragmatic supplementation with infant formula was reported when maternal physiological parameters (lactation volume or postpartum recovery status) proved inadequate to meet neonatal nutritional requirements. Notably, three modifiable barriers emerged: psychological distress, sleep fragmentation secondary to nocturnal feeding demands, and occupational reintegration pressures. Fathers’ support experiences were shaped by how they perceived and responded to challenges faced by their partners, primarily perceived insufficient milk supply and maternal fatigue. Maternal constraints served as critical catalysts that defined the paternal support experience, prompting emotional engagement and comanaging behaviors.

F3: Keeping up with breastfeeding takes a real physical toll, especially because it messes up your sleep with feedings at all hours of the night.

F6: The hardest part is definitely the broken sleep, having to wake up multiple times at night.

F1: When the feeding doesn’t seem to be going well—like if the baby might not be getting enough—it makes us worried and anxious. When there are difficulties with breastfeeding, we might choose to give some formula instead to solve the problem right away.

F4: (My wife’s) milk supply wasn’t great, so we had to add formula to make sure the baby got enough nutrition and was growing well.

F8: Going back to work made it harder to keep up with breastfeeding, especially when my wife wasn’t feeling well.

#### 3.3.2. Paternal Participation Barriers: Temporal and Energetic Constraints

Work‐related time and energy constraints were the central, interlinked barriers to fathers’ practical involvement in breastfeeding support. Insufficient time, such as demanding employment schedules and a lack of workplace flexibility, directly limited their physical presence during feeding times. Furthermore, this work burden was frequently described as depleting mental energy and exacerbating fatigue, thereby reducing their capacity for the attentive, patient engagement required for effective breastfeeding support (e.g., positioning assistance and infant soothing).

F1: After I went back to work, I had much less energy for childcare, especially for helping with the baby at night.

F12: Juggling work and childcare left me with almost no personal time at all. In a way, it made using the formula seem like an easier choice.

#### 3.3.3. Intergenerational Support Mechanisms

Multigenerational kinship networks exerted substantial influence through two pathways: (1) provision of culturally embedded lactation knowledge (e.g., galactagogue dietary practices) and (2) modeling of positive breastfeeding behaviors. This intergenerational transfer of embodied knowledge significantly enhanced paternal self‐efficacy and engagement.

F3: The older generation helped by preparing traditional foods that are believed to help with milk production, and they also encouraged the idea that breastfeeding is the right thing to do.

F12: Seeing relatives (sister) breastfeed their babies for a long time (over a year) gave us both a practical example and motivation.

### 3.4. Macrosystem: Incomplete Sociocultural Support Systems

#### 3.4.1. Evolving Sociocultural Perceptions of Paternal Caregiving Roles

Widespread societal endorsement of breastfeeding reinforces parental caregiving behaviors, facilitating its broader adoption. Concurrently, shifting cultural norms are redefining paternal roles, encouraging more active involvement in infant care. Fathers in this study frequently noted their awareness of broader societal changes regarding paternal roles. This subtheme presents their descriptions of these macrolevel sociocultural contexts as external forces they perceived. Most fathers now recognize breastfeeding as a shared familial responsibility rather than a maternal duty alone, acknowledging its physical demands and proactively seeking relevant knowledge. However, initial paternal engagement is often hindered by limited expertise, resulting in transitional uncertainty.

F1: Fathers need to learn about this stuff too. I use little bits of free time to watch short videos on parenting that are based on evidence.

F8: I consult medical professionals’ public accounts (e.g., Dingxiang Doctor) and user‐generated guides on lifestyle platforms.

F7: We looked at the posters in the hospital and set a goal for how long to breastfeed, but we’re still not sure about the specific steps to make it work.

#### 3.4.2. Gaps in Institutional Breastfeeding Support Frameworks

Multisectoral efforts (healthcare institutions, clinicians, and digital media) actively promote breastfeeding benefits. Fathers reported enhanced confidence through hospital‐distributed materials, instructional videos, and antenatal seminars. Digital ecosystems (e.g., Bilibili and Rednote) further provide peer‐driven knowledge repositories and troubleshooting networks. Nevertheless, systemic deficiencies persist in paternal‐centric resources, with current educational content predominantly maternal focused.

F3: Existing campaigns ubiquitously extol breastfeeding’s merits yet fail to operationalize paternal roles. A paradigm shift toward father‐inclusive instructional media—demonstrating concrete support modalities—would significantly enhance utility.

F12: Healthcare systems must optimize information accessibility by leveraging youth‐engaged platforms (e.g., video‐sharing sites, forums) and streamlining knowledge acquisition pathways.

### 3.5. A Typical Case: The Interaction of Micro, Meso, and Macro Factors in One Father’s Experience

To systematically illustrate how multilevel factors interact in an individual father’s experience, we present the case of Father F12 (aged 40, master’s degree, clerk, and first‐time father). Although he had attended breastfeeding training (Table 1), he still reported low self‐efficacy in practice.

At the microsystem level, despite attending training, F12 admitted that his theoretical knowledge did not translate into practical confidence. He reported: “I read some online articles and went to a class, but when the baby cried and would not latch, I felt completely helpless—I just stood there.” This gap between knowledge and hands‐on ability led him to avoid direct assistance during feeding.

At the mesosystem level, his wife experienced hypogalactia and sleep deprivation, which increased F12’s caregiving burden. His employer did not offer flexible paternity leave, so he returned to work 5 days postpartum. He stated: “Juggling work and childcare left me with almost no personal time. In a way, it made formula seem like an easier choice.” His mother‐in‐law, who lived with them, provided traditional lactation advice but criticized his lack of involvement, creating family tension.

At the macrosystem level, F12 was aware of national breastfeeding promotion campaigns but perceived them as targeting mothers only. He noted: “All the posters in the hospital were about what mothers should do. There was nothing for fathers.” This lack of father‐inclusive public messaging reinforced his passive role.

This case demonstrates how even with training, microsystem low self‐efficacy, mesosystem work‐family conflict, and macrosystem policy neglect jointly shaped a pattern of minimal paternal breastfeeding support. It also illustrates the need for coordinated interventions across all three levels.

## 4. Discussion

Guided by the SESs theory, our findings reveal that the experiences of newborn fathers in supporting breastfeeding are shaped by a complex interplay of factors across individual (micro‐), interpersonal/organizational (meso‐), and societal/cultural (macro‐) levels. The following discussion interprets these findings through this multilevel lens, elucidating how dynamic interactions across these systems collectively influence paternal support behaviors.

### 4.1. Microsystem: Bridging the Father’s Attitude–Behavior Gap and the Role of Personal Resources

At the individual microsystem level, this study identified a prevalent positive attitude toward breastfeeding among new fathers, yet a marked deficiency in their practical support behaviors, primarily stemming from low self‐efficacy. This knowledge–action gap is a critical barrier, consistent with Krikitrat et al.’s [18] qualitative study on Thai fathers, further confirming that low paternal breastfeeding self‐efficacy is a common cross‐cultural phenomenon. While public education has enhanced awareness, as noted in prior studies [19, 20], it fails to equip fathers with the competence needed for hands‐on support. Fathers’ anxiety often arises from a lack of practical skills in positioning, latching, and problem‐solving, making them feel inadequate during actual feeding. Beyond general knowledge, a father’s prior parenting experience (e.g., F2, F3, F6, F9, and F13) constitutes a key personal resource within his microsystem. This lived experience can enhance practical confidence, contrasting with the “trial‐and‐error” approach described by first‐time fathers. This individual‐level factor (self‐efficacy and experience) is profoundly influenced by and interacts with outer systems.

Notably, only two fathers (F10 and F12) reported having attended any form of breastfeeding or parenting training. Training serves as a vital pathway for fathers to acquire crucial hands‐on support experience, which forms the foundation for enhancing their self‐efficacy. This lack of training may be a significant contributing factor to the prevalent low self‐efficacy among newborn fathers identified in this study. Therefore, interventions at this level should transcend knowledge dissemination to include practical, hands‐on skill training (e.g., simulation‐based workshops) and continuous, accessible support (e.g., peer groups, hotlines) to directly build individual capability and bridge the attitude–behavior gap.

### 4.2. Mesosystem: The Interplay of Family, Workplace, and Institutional Support

The mesosystem, encompassing the immediate social and organizational networks, presented both barriers and facilitators. Within the family unit, a key interaction was observed: maternal constraints (e.g., hypogalactia and fatigue) directly triggered shared family coping strategies, such as formula supplementation (F1 and F4). This illustrates how a microsystem challenge (maternal physiology) precipitates a mesosystem behavioral response (joint parental decision‐making). Furthermore, the attitudes and practical support from grandmothers exerted significant influence, potentially reinforcing or undermining the father’s supportive role [21, 22]. The influence of grandmothers as part of the family mesosystem is supported by Negin et al.’s systematic review [23]. Our findings further nuance this by revealing that inconsistent messaging among father, mother, and grandparents creates confusion and undermines paternal confidence—a triadic dynamic rarely captured in previous studies. The family mesosystem dynamics differ for fathers with more than one child, where existing routines and sibling dynamics add complexity but may also offer experiential leverage.

Concurrently, the workplace (exosystem) strongly interacted with the family mesosystem. Paternal time and energy constraints, dictated by occupational demands and insufficient leave policies, directly reduced their capacity for engagement in domestic support (F1 and F12). This creates a conflict between the mesosystems of family and work, straining the father’s role. Healthcare institutions, another critical mesosystem component, provided foundational information but often failed to offer father‐inclusive, practical guidance (F3 and F12). This represents a missed link between institutional support and individual father needs. Thus, optimizing this level requires enhancing intrafamily communication to align support goals, implementing family‐friendly workplace policies (e.g., flexible paternity leave), and transforming healthcare services to actively include and skill‐build fathers, thereby strengthening the supportive connections between these intermediate systems.

### 4.3. Macrosystem: Shaping Norms and Systems for Sustainable Change

At the broadest macrosystem level, evolving sociocultural perceptions and institutional frameworks set the overarching context. There is growing societal endorsement for active fatherhood and breastfeeding, which positively influences paternal role recognition. However, deep‐seated norms still often view infant feeding as a maternal domain, limiting the perceived legitimacy of fathers’ involvement. This macrolevel culture subtly shapes attitudes within families (mesosystem) and individual fathers’ self‐perception (microsystem). Comprehensive breastfeeding support systems can directly contribute to achieving the UN Sustainable Development Goals, including gender equality, women’s rights, and economic growth [24]. International research has systematically examined breastfeeding support systems at national, environmental, and individual levels [25–27]. While this provides a universal framework, within the specific sociocultural context of China, the expression and navigation of these macrosystemic influences take on distinct characteristics. Within the family mesosystem, intergenerational support manifests through the transfer of traditional lactation knowledge and motivational modeling, while an evolving paternal role is evidenced by the proactive pursuit of parenting knowledge via digital platforms. This interplay between tradition‐based and contemporary support‐seeking occurs against the backdrop of a historic policy shift, collectively redefining expectations. Consequently, despite international models and global goals, developing culturally appropriate support systems remains a pressing challenge in China [28].

Systemically, while national policies like the Breastfeeding Promotion Action Plan (2021–2025) [6] set ambitious targets, their translation into concrete, father‐sensitive practices remains inadequate. Nyaaba et al.’s content analysis of breastfeeding policies further found that only about 68% of breastfeeding‐only policies mentioned fathers, and fewer than half of those mentions were accompanied by explicit strategies to involve fathers [29]. Support infrastructures, such as lactation rooms and public campaigns, are often designed with mothers as the primary audience. This macrosystemic oversight filters down, resulting in mesosystemic institutions (hospitals and media) producing content that fails to engage fathers effectively. Therefore, advancing support requires concerted macrosystemic actions: (1) strengthening sociocultural guidance through media to model and normalize active paternal support; (2) enhancing public services by ensuring policies mandate and fund father‐inclusive education and infrastructure; and (3) advocating for lactation‐supportive workplace regulations aligned with national development goals. These efforts can reshape the broader environment to enable and sustain changes at the meso‐ and microlevels.

### 4.4. Interplay Across Ecological Systems: A Synergistic Perspective

This study reveals that, within the routine postpartum ecology, paternal support experiences are shaped by the complex interplay of factors across the micro‐, meso‐, and macrosystems. This finding presents a theoretically insightful contrast with research conducted in the Neonatal Intensive Care Unit context, such as that by Li et al. [12]. In the NICU, a medical crisis triggers a contraction of the ecosystem towards the meso (healthcare) system, where the father’s experience becomes predominantly defined by medical protocols and institutional workflows. In contrast, the ecosystem navigated by fathers in the present study exhibits a more complete and dynamically balanced multilevel configuration. For instance, a father’s low self‐efficacy (microsystem) can be exacerbated by conflicting advice from family elders (mesosystem), which is itself rooted in traditional cultural norms (macrosystem). Conversely, a supportive workplace policy (exosystem/macrosystem) can increase a father’s available time, enhancing his presence and engagement at home (mesosystem), which in turn allows for skill practice that boosts his confidence (microsystem). These dynamic interactions underscore that effective intervention necessitates a synergistic approach—simultaneously building individual paternal skills (micro), fostering supportive family and work environments (meso/exo), and advocating for enabling policies and cultural shifts (macro). This multilayered strategy is essential to bridge the identified attitude–behavior gap, alleviate participatory burdens, and ultimately promote broader breastfeeding success through competent and engaged paternal support.

## 5. Limitations

This study has several methodological and contextual limitations. First, the exclusive focus on urban tertiary hospitals limits generalizability to rural or primary care settings where resource disparities may significantly alter paternal engagement dynamics. In rural areas or primary care facilities, where access to specialized lactation support and educational materials may be limited, paternal challenges and support strategies could differ substantially. Second, the participants were primarily urban, well‐educated fathers, which may limit the transferability of findings to rural populations or fathers with different socioeconomic backgrounds. The challenges and coping strategies identified may be specific to this demographic. Third, reliance on self‐reported data from 12 paternal participants introduces potential recall and social desirability biases, particularly regarding self‐efficacy assessments. Future research should purposively sample more diverse populations to enhance the theoretical transferability and contextual range of the findings.

## 6. Conclusion

This social–ecological study reveals that paternal support for breastfeeding is characterized by positive attitudes yet critically undermined by low self‐efficacy, resulting in insufficient practical engagement. The observed behavioral patterns emerge from dynamic interactions across multiple levels: individual knowledge–attitude constructs, mesosystemic family/workplace supports, and macrosystemic sociocultural–public service infrastructures. To effectively bridge this attitude–behavior gap, a comprehensive intervention framework is proposed—integrating enhanced practical skill training, sustained support systems, optimized familial communication, workplace accommodations, sociocultural norm restructuring, public service improvements, and targeted paternal role advocacy. These synergistic strategies collectively reduce participatory barriers while elevating engagement quality, ultimately catalyzing broader breastfeeding adoption through empowered paternal involvement.

## 7. Implications for Nursing Management

This study’s socio–ecological findings necessitate transformative nursing management strategies to bridge the paternal attitude–behavior gap in breastfeeding support. At the clinical level, nursing leaders should champion the development and integration of standardized, couple‐based educational modules into antenatal and postnatal care, equipping fathers with practical skills and legitimizing their role. Concurrently, implementing “father‐friendly” clinical protocols is crucial, such as designated support liaisons, direct inclusion in consultations, and tailored discharge planning. To sustain engagement, nursing services should establish dedicated postdischarge support channels. At the organizational level, nursing administrators must advocate for family‐friendly workplace policies, partnering with employers to implement flexible work arrangements and fully utilize paternity leave, in alignment with national family development goals. They must also optimize hospital infrastructure to support father inclusion. Professionally, nursing organizations should leverage their collective voice to shape sociocultural norms through positive media messaging and to influence public policy, advocating for stronger enforcement of supportive regulations (e.g., lactation rooms) and contributing to national care standards that recognize paternal engagement as a quality indicator. Through these concerted actions across practice, organization, and advocacy, nursing can transform care delivery and systemic environments, empowering fathers as confident partners to improve breastfeeding outcomes.

## Author Contributions

Siya Lin was responsible for conceptualization and methodology, data curation, prepared the original draft, and revised the manuscript; Yacui Huang conceptualized the study and was responsible for the interview, reviewed the manuscript; Shunmei Chen was responsible for conceptualization, data analysis, discussion, and revision of the manuscript; Minyan Wu and Haiai Long were responsible for data analysis, discussion, and revision of the manuscript; Dan Tian was responsible for conceptualization, resources, supervision, project administration, and revision of the manuscript. Siya Lin, Yacui Huang, and Shunmei Chen contributed equally to this work and share first authorship.

## Funding

This study was supported by the Guangdong Provincial Medical Science and Technology Research Fund Project in 2023 (B2023110).

## Disclosure

All authors have read and approved the final version for submission.

## Ethics Statement

This study was approved by the Ethical Committee of Zhujiang hospital ethics committee (2022‐KY‐234‐01).

## Conflicts of Interest

The authors declare no conflicts of interest.

## Data Availability

The data that support the findings of this study are available on request from the corresponding author. The data are not publicly available due to privacy or ethical restrictions.
